# An Ishmaelite

**Published:** 1888-09-22

**Authors:** Leslie Keith

**Affiliations:** Author of "The Chilcotes," "Uncle Bob's Niece," "East and West," etc.


### AN ISHMAELITE.

By LESLIE KEITH,

Author of "The Chilcotes," "Uncle Bob's Niece," "East and West," etc.

*(Continued from p. 394.)*

### CHAPTER XXV.—TREMBLING IN THE BALANCE.

It was thus calamity, and not love, that brought Delia back to her own people.

Arthur had left town early, at his uncle's request, on business that would detain him a day and night, and the tidings of the disaster escaped his notice till evening, when he chanced to light on the account of it given in the daily print. He was then too far from London to obey his instinctive impulse to rush back there at once. Chafe and fret as he might, he had to finish the task he had undertaken, and to rein in his devouring impatience till it was accomplished, and he was free once more.

Thus Delia, whom a considerate neighbour had summoned, was the first to reach the familiar street where there was no longer any home to welcome her.

By the strenuous efforts of the fire brigade, the destruction had been mainly confined to the two shops and the tenement above them which Proudie owned. But the wreck there was complete, and a gaunt, desolate ruin, with bulging walls and gaping windows, faced the trembling girl. Delia gave scarcely a glance to the melancholy spectacle. Her crowding emotions were rushing on to the meeting in store for her; a sick dread clutched at her heart, but she dared not put it in words; she had heard a name spoken in a hushed whisper when the compassionate neighbours came out to greet her: her father and mother, her aunt and sister were safe, she knew, but there was some one else—she could not ask a question.

"I want Barbara," she said, looking up with a piteous appeal into a good-natured woman's face; "take me to her."

"Your ma and pa's in there, my dear," said the friend thus appealed to, pointing with an upward thumb to the tinsmith's house. The tinsmith had suffered too, in scorched and blackened walls and broken windows, but he had generously extended his hospitality to his neighbours in their need. "You'll go to your pore ma, Miss Delia?"

"Take me to Barbara," said Delia.

They led her to a house a little higher up the street, where Barbara was keeping watch in a sick-room.

"They say he'll die, pore gentleman; he's burned that bad the doctors can't do nothink for him," said one, with that curious bluntness of feeling humbler folks always display when discussing the chances of life or death.

Delia broke from them, and dragged herself trembling up the long stairs.

"At the top of the 'ouse—the back room, dearie," someone called after her, for her guidance; "the back room, where it's quieter-like."

Delia took that sick horror of hers up with her step by step: it was a wildly foolish fear that devoured her, but she could not reason with it or control it. She had nearly reached the top when a door opened there and Barbara came out. Delia looked at her with a pallid, dumb wretchedness that the elder girl somehow understood.

"No, dear Del," she said, taking the trembling little sister in her arms. "Do not be afraid; he is safe and well. He is not here. He has not been here at all."

The reaction was too much. Delia clung to her sister and began to sob hysterically.

"Hush," said Barbara, with a backward glance at the room she had left. "You must be brave, Delia, for my sake." She drew her into an adjoining room and soothed her.

"The paper said—it was Mr. Arthur Eastgate," sobbed Delia.

"No, Del, it was a mistake; he has not been here for two days; I think he cannot know. You will be happy, dear little sister." She put her head down on Delia's shoulder, yielding for a moment to her weakness.

"You will have your heart's desire; he has never wavered."

"Oh, Barbie," whispered Delia, repressing the tide of joy that was surging in her heart, and thinking of the sorrow that had passed her by. "Then it was his cousin?"

"Yes, it was his cousin."

"He will not die—he who was so splendidly brave?"

"I do not know," said Barbara, lifting a sad, composed face. "I pray God he may be spared; but I have little hope. He gave himself for us, Del, but it was to save George Tucker he went that last time."

"Oh!" cried Del, fiercely; "to save him!"

"Don't say that, Delia." Barbara looked at her with a dilation and flash of the grey eyes. "It was a fine thing to do, and if there were other things in his life that were less noble, surely this may help to wipe away the debt. I cannot stay with you, Del, I must go back."

"Let me help you—I will be very calm and quiet; let me help you, Barbara."

There was no lack of willing service on Fred's behalf. The story of his bravery had reached all ears, and the little street was proud of its hero. The gentleman whom it had looked on with distrust and suspicion was now the favourite of the hour. Honest English hearts always find applause for a courageous act, and Barbara might have had the help of every matron in the street if she had cared to make such a claim. But she jealously excluded everyone and kept watch alone, except when she was persuaded to let her sister or aunt relieve her.

"It was for us he suffered," she said, "and you would have him sent to a hospital, and given over to the care of strangers! Who has a better right to nurse him than I?"

She gave not a thought to convention; and, indeed, Mrs. Grundy, in matters of this kind, is less of a power in Gillespie Street than she is in some other quarters, and no one blamed Barbara for giving her best devotion in payment of Fred's debt. If there were some who guessed her secret, they found that natural too.

When Arthur saw Delia again, it was by Fred's sick-bed. They met quite simply; Arthur took the little cold hands in his and looked into the sweet, shy eyes, but no word of love passed his lips.

It was no time for joy while Fred's life hung in peril. Would he pull through and be given back to them? Many heartfelt prayers went up on his behalf. Honest Proudie was more concerned over his condition than over the losses he had sustained, though these as good as ruined him. Miss Barbara guarded his sick-room with a fierce, unsparing watchfulness.

The news had spread to other quarters too, and an old man sitting lonely in the big, dull library at Portland Place, would draw out with a shaking hand a fragment cut from the newspaper, and read it over and over with lips that trembled.

We are apt, perhaps, to lay too much stress on one brave act done under the compulsion of overmastering emotion, and to think that it ought to hallow the years in which the soul has consented to baseness: even the weakest can be heroic at a supreme crisis, but the heroism that ennobles the daily path of common life and is strong to endure and overcome, though there is no one to applaud, is of another growth. Fred was not of the stuff of which such conquerors are made; but who that knew the history of his fall would grudge him this one redeeming chance?

Not the old father, his pride broken down at last—listening with outward sternness but inward dread for Arthur's step and the tidings he brought—not Arthur; not, oh, most of all, not Barbara.

But there were others who took what is called the common-sense view of the matter, and who thought it would have been rather more comfortable for all concerned if Fred had gone the whole way of martyrdom and allowed himself to be burned completely, instead of crawling back to life and becoming once more a family perplexity.

In the spring of that year Nellie had a serious illness, and his anxiety for her and fear of losing her, softened her husband's mood towards her, and arrested the progress of their estrangement. Weak, foolish Nellie came back to shelter herself under her husband's kindness once more, and in the revulsion of her feeling towards him, she told him everything.

She was very fragile yet, and Bellynge had been warned that he must be specially gentle and considerate.

"You should have told me, Nellie," was all he allowed himself to say by way of reproach. "A husband doesn't care to have a secret like that sprung on him. How was I to know that you had cared for the fellow before you took me?"

"I was so miserable then, and I thought you would not understand. But it is all over—all over," she said, "and I think, Richard it was not so much that I cared for him, but that you were cold and stern, and you frightened me."

Poor Nellie! He took her hand, and looked at her with a kind of puzzled pity and wonder. There was very little in his own nature that corresponded to hers or helped him to understand it.

"And Fred had stopped caring for me," she went on. "I saw it that day—that day I went to see him. It was that girl he cared for, and not me; that girl in the shop."

Nellie was too true a woman not to harbour some jealous feelings towards her rival.

"Well," said Bellynge with a resigned sigh, "I suppose he'll always be a thorn in the flesh. It would have been more decent if he had died, when he was about it. He has just done enough to remind the public of him—to stir the mud, and set chatter going; whereas, if he had died, we might have set him up as a martyr, and buried his follies with him."

"Arthur will be glad," murmured Nellie, her conscience stirring her. "I don't like you to wish he had been killed, Richard."

"My wishes won't mend the matter," said Bellynge with a shrug. "We've got to put up with him, I suppose, since he's made up his mind to live. And now he'll marry this girl, and start an eating-house on his own account, no doubt, with Arthur as partner, unless your uncle forgives the prodigal and takes him back?"

"I don't think Uncle Joseph will do that, and I am sure Fred wouldn't return to Portland Place. He will marry Barbara Proudie, and we shall never see him any more."

"That we certainly shall not, if I can help it," said Richard, with good-humored firmness. "We can't afford to be mixed up with people like that, Nellie. There, my dear, you mustn't talk any more: go to sleep and get well as fast as you can, for I've promised to take you down to Christina as soon as you can travel."

But though Nellie's vanity had been scourged, the old lingering affection was not yet quite dead in her heart.

"Poor Fred," she said; "poor Fred! Oh, Richard, I wish you had known him before—before all those dreadful things happened to him. You would have understood better how easy it was to care for him then, Richard."

*(To be continued.)*

